# Preferred Mode of Atmospheric Water Vapor Condensation on Nanoengineered Surfaces: Dropwise or Filmwise?

**DOI:** 10.1021/acs.langmuir.3c00022

**Published:** 2023-04-04

**Authors:** Tibin M. Thomas, Pallab Sinha Mahapatra, Ranjan Ganguly, Manish K. Tiwari

**Affiliations:** † Department of Mechanical Engineering, Indian Institute of Technology Madras, Chennai 600036, India; ‡ Department of Power Engineering, Jadavpur University, Kolkata 700106, India; ¶ Nanoengineered Systems Laboratory, UCL, London WC1E 7JE, U.K.; § Wellcome/EPSRC Centre for Interventional and Surgical Sciences, UCL, London W1W 7TS, U.K.

## Abstract

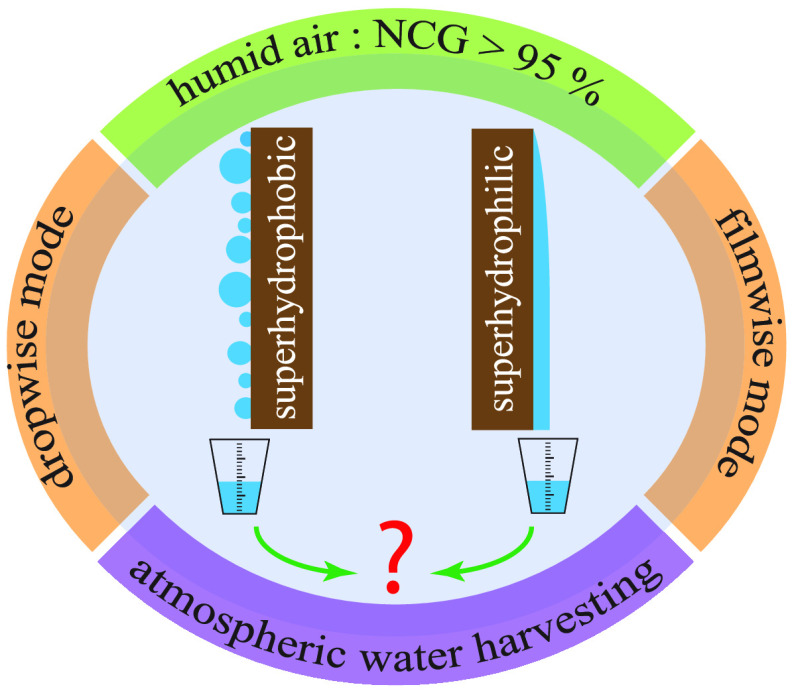

Condensing atmospheric water vapor on surfaces is a sustainable approach to addressing the potable water crisis. However, despite extensive research, a key question remains: what is the optimal combination of the mode and mechanism of condensation as well as the surface wettability for the best possible water harvesting efficacy? Here, we show how various modes of condensation fare differently in a humid air environment. During condensation from humid air, it is important to note that the thermal resistance across the condensate is nondominant, and the energy transfer is controlled by vapor diffusion across the boundary layer and condensate drainage from the condenser surface. This implies that, unlike condensation from pure steam, filmwise condensation from humid air would exhibit the highest water collection efficiency on superhydrophilic surfaces. To demonstrate this, we measured the condensation rates on different sets of superhydrophilic and superhydrophobic surfaces that were cooled below the dew points using a Peltier cooler. Experiments were performed over a wide range of degrees of subcooling (10–26 °C) and humidity-ratio differences (5–45 g/kg of dry air). Depending upon the thermodynamic parameters, the condensation rate is found to be 57–333% higher on the superhydrophilic surfaces compared to the superhydrophobic ones. The findings of the study dispel ambiguity about the preferred mode of vapor condensation from humid air on wettability-engineered surfaces and lead to the design of efficient atmospheric water harvesting systems.

## Introduction

The dwindling reserve of potable water on the planet as a result of industrialization, climate change, and environmental pollution is a major challenge in the coming decades. Recently, it has been estimated that nearly half a billion of the global population is facing severe water scarcity throughout the year, while about four billion people are facing the same for at least one month of the year.^1^ Capturing water vapor from the humid air by condensation is a sustainable water-production technology, particularly in arid environments, to address this potable water shortage.^2−4^ The conversion of water vapor, abundantly present in the atmospheric air, into liquid phase is achieved by cooling the air below dew point. It begins with the formation of liquid nuclei on the surface once its temperature falls below the saturation temperature. The surface can be cooled using the contact approach by connecting to a thermoelectric cooler or by circulating cold refrigerant fluid. These technologies are less energy-efficient and require a substantial quantity of electrical energy for cooling. In contrast, radiation cooling is a promising passive technology for cooling the surface by emitting heat via thermal radiation without the need of electrical energy.^5,6^ However, radiative cooling is less effective during the day, and many researchers are actively attempting to improve it through the discovery of novel materials.^7,8^

In the earth’s atmosphere, the mass fraction of water vapor is always below 5%, with more than 95% being noncondensable gases (NCGs). Condensation of the vapor requires a subcooled surface for both pure vapor and vapor in humid air. The mechanism of vapor condensation in the presence of NCGs is distinct than pure vapor condensation due to the formation of thermal and concentration boundary layers outside the liquid–vapor interface, which are not observed during pure vapor condensation. Along with the growth of liquid nuclei, the NCG molecules present in the humid air are accumulated over the interface of the nuclei and form an NCG diffusion layer.^9,10^ This layer acts as a barrier to water vapor diffusion from the bulk humid air to the interface, and even a small quantity of NCG (mass fraction of around 0.5%) can reduce heat transfer rates by 50% when compared to a pure vapor environment.^11,12^ Furthermore, depending on the water vapor concentration, the condensation heat transfer rate in humid air can be 2–3 orders of magnitude lower than that in pure vapor.^13,14^ Prior research over the past century has shown that the condensation heat transfer rate of the dropwise condensation (DWC) mode can be 1 order of magnitude higher than the filmwise condensation (FWC) mode under pure vapor conditions.^15−17^ However, it remains unclear which mode of condensation is better for water harvesting from humid air, despite decades of research.

Over the last two decades, advances in surface chemistry and micro/nanofabrication techniques have inspired researchers to custom-engineer the wettability of the condensing surface for better condensation effectiveness, which is required for water-energy nexus applications.^18−25^ Dropwise condensation on a superhydrophobic surface has been proposed for water harvesting in several publications due to the early departure or enhanced mobility of condensing droplets, allowing greater access to the dry condensing area for continuous nucleation. In addition, filmwise condensation on superhydrophilic surfaces has also been proposed for water harvesting. Table 1 summarizes the research articles that reported the overall condensation rate on the nonwetting superhydrophobic and wetting superhydrophilic surfaces in the atmospheric humid air conditions. Clearly, the majority of the works cited in Table 1 report that filmwise mode is more effective than dropwise mode for humidity harvesting.^26−35^ However, these observations did not receive comprehensive attention and, as a consequence, have remained a mystery. This result is contradictory to the general perception about the heat transfer efficiency of dropwise and filmwise modes of condensation under pure vapor conditions, but a plausible reason has not yet been addressed. Furthermore, because these investigations are limited to a narrow range of ambient conditions, making a firm conclusion about the most effective mode of condensation in humid air is difficult from Table 1.

**Table 1:** Summary of Previous Studies Reporting the Overall Mass Flux during Filmwise Condensation (FWC) on Superhydrophilic Surfaces and Dropwise Condensation (DWC) on Superhydrophobic Surfaces in Vertical Orientation^a^

ref	substrate type and contact angle	surface morphology	environmental condition	subcooling Δ*T* (°C)	difference in humidity ratio Δω (g/kg)	percentage change in FWC mass flux with DWC (%)
(26)	silicon, θ = 161°	TiO_2_ nanorods	*T* = 25 °C, RH = 93.5%	20.87	14.06	+109
(27)	aluminum, θ = 161.2°	hierarchical Al_2_O_3_	*T* = 20 °C, RH = 80%	14.15	7.24	+50
(27)	aluminum, θ = 161.2°	hierarchical Al_2_O_3_	*T* = 35 °C, RH = 80%	8.6	11.84	+37.5
(28)	copper tube, θ = 161.7°	CuO nanostructures	*T* = 40 °C, RH = 80%	32.5	33.69	+4.5
(29)	copper foil, θ = 168°	CuO nanostructures	*T* = 26 °C, RH = 50%	12.8	6.13	+26.4
(30)	steel tube, θ = 159°	carbon nanotubes	*T* = 40 °C, RH = 80%	31.9	33.48	–29.4
(31)	steel tube, θ = 169°	carbon nanotubes	*T* = 30 °C,T RH = 80%	14.2	12.85	–34.4
(32)	aluminum, θ = 160°	hierarchical Al_2_O_3_	*T* = 24.5 °C, RH = 70%	13.7	8.11	+21
(32)	aluminum, θ = 160°	hierarchical Al_2_O_3_	*T* = 24.5 °C, RH = 80%	15.8	10.08	–1.2
(32)	aluminum, θ = 160°	hierarchical Al_2_O_3_	*T* = 24.5 °C, RH = 90%	17.7	12.07	–27.8
(33)	aluminum, θ = 142°	hierarchical Al_2_O_3_	*T* = 14 °C, RH = 100%	9	4.57	+78.5
(34)	silicon, θ = 162°	rough nanostructures	*T* = 25 °C, RH = 92.5%	13.69	10.91	–12.1
(35)	aluminum, θ = 148°	hierarchical Al_2_O_3_	*T* = 23.6 °C, RH = 50%	8	5.25	+8.9

a The reported results are too dispersed and cannot be correlated with operating conditions such as degree of subcooling or humidity ratio difference. The influence of several experimental uncertainties, such as dynamic changes in relative humidity within the condensation chamber, perturbation or velocity of the condensing fluid, edge effects of the condensing surface, surface aspect ratio, and so on, could be implicated for the inconclusive trend. The compiled data reveals a lack of understanding regarding the overall impact of wettability modification on water yield during humid air condensation. The reported contact angle of the water drop on the superhydrophobic substrate is mentioned, and the contact angle on the superhydrophilic substrate was below 5°.

To bridge this gap, we performed the experiments on two superhydrophobic and two superhydrophilic surfaces under humid air conditions in a much more comprehensive range of degrees of subcooling (10–26 °C) and humidity ratio differences (5–45 g/kg of dry air). The humidity ratio difference (Δω) is defined as the difference between the specific humidity of the bulk humid air and the saturated vapor at the substrate temperature conditions. We observed that, depending on the specific combination of the experimental conditions investigated, filmwise mode on superhydrophilic surfaces can provide 57–333% greater condensate yield than the dropwise mode on superhydrophobic surfaces. To substantiate these observations, a detailed assessment has been performed using basic condensation heat transfer theory for computing the thermal resistance offered by coating layer, condensate, liquid–vapor interface, diffusion layer, etc.^36,37^ This shows that, in the presence of NCGs (specifically above 95%), the thermal resistance across the condensate and coating layer becomes negligible, and vapor diffusion becomes the dominant mechanism. We also investigate the different rivulet structures of the condensate forming on superhydrophilic surfaces due to the surface morphology, spreading dynamics, roughness, etc., and provide first insights into how they influence the condensation rate in a humid air environment. Surprisingly enough, the superhydrophilic surface with vertical rivulet channels outperformed those with branched rivulet channels by 10–20%. The current study also sheds light on the previously unknown benefits of the filmwise mode in humid air, which have far-reaching implications for developing efficient engineered interfaces for humidity harvesting.

## Results and Discussion

### Surface Morphology

**Figure 1:**
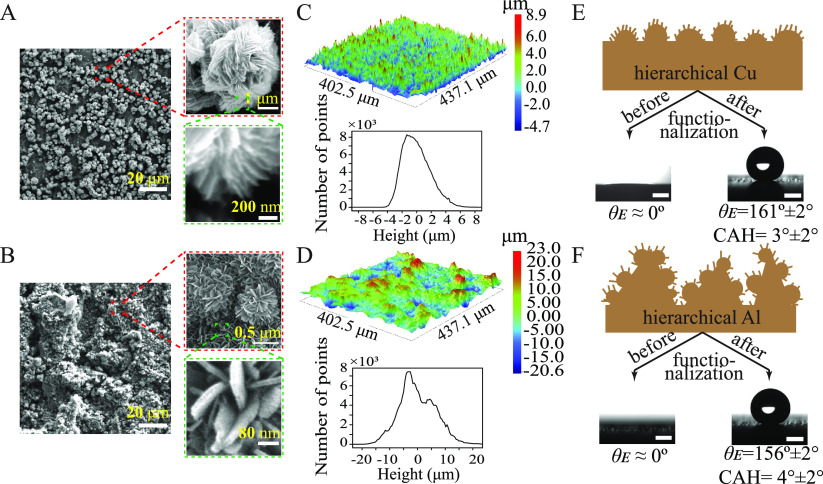
Surface topography and characterization. SEM micrograph of micro/nano textured (A) copper and (B) aluminum substrate at various resolutions. Profilometer micrograph of (C) copper and (D) aluminum substrates. The frequency distribution graph depicts the number of peaks and valleys at each bin size. The schematic illustration of the randomly generated micro/nano textures on (E) copper and (F) aluminum substrates. The scale bars in contact angle images are 1 mm.

To investigate the condensation performance on completely wetting and nonwetting surfaces under humid air conditions, hierarchical surfaces of copper and aluminum were fabricated. Figure 1A and B shows the surface morphology of the micro/nano textured copper and aluminum substrates, both of which exhibit superhydrophilic nature. The hierarchically rough substrates were functionalized with perfluoroocty-triethoxysilane (PFOTES) to achieve superhydrophobicity. The surface morphology of the copper and aluminum superhydrophobic surfaces was similar to the corresponding superhydrophilic surfaces since the self-assembled monolayer of PFOTES does not modify the hierarchical structures.^38^ The copper substrate consists of microflower shaped cupric oxide structures ranging in size from 2–4 μm, that are orderly arranged on the surface to some extent (Figure 1A).^39,40^ The order of the structures can be visualized in the profilometer micrograph, as shown in Figure 1C. The spacing between the microflowers was in the range of 0.5–15 μm. Conversely, the surface features of the aluminum sample were random and consisted of microbumps of different sizes, as shown in Figure 1B and D. The height and width of these microbumps were in the range of 5–30 μm and 5–60 μm, respectively. Flower-shaped features of size 0.5–1 μm are found to surround the microbumps (Figure 1B). The microflower structures on the copper substrate consist of thin sheets of nanostructures, and the aluminum substrate consists of blade-shaped nanostructures.

The actual surface topography of the copper and aluminum substrates can be schematically represented based on the SEM and profilometer micrographs in Figure 1E and F, respectively. The average roughness of the modified copper and aluminum substrates was *R*_a_ = 1.5 μm, *R*_a_ = 5.3 μm, respectively. The frequency distributions in Figure 1C and D show that ∼99% of the peaks and valleys lie in the range of ±4 and ±15 μm for copper and aluminum substrates, respectively. These indicate that the depth of the textures is higher on the aluminum substrate than on the copper substrate. Prior to PFOTES functionalization, the contact angle of the aluminum and copper substrates was nearly zero, and a water drop dispensed on the substrate completely spread on the surface through hemiwicking. The equilibrium contact angle of the PFOTES-coated copper surface was θ = 161 ± 2°, and the contact angle hysteresis (CAH) was 3 ± 2°. The equilibrium contact angle of the superhydrophobic aluminum substrate was θ = 156 ± 2° and CAH = 4 ± 2°.

### Microscale Condensation Behavior on Superhydrophobic Surfaces

**Figure 2:**
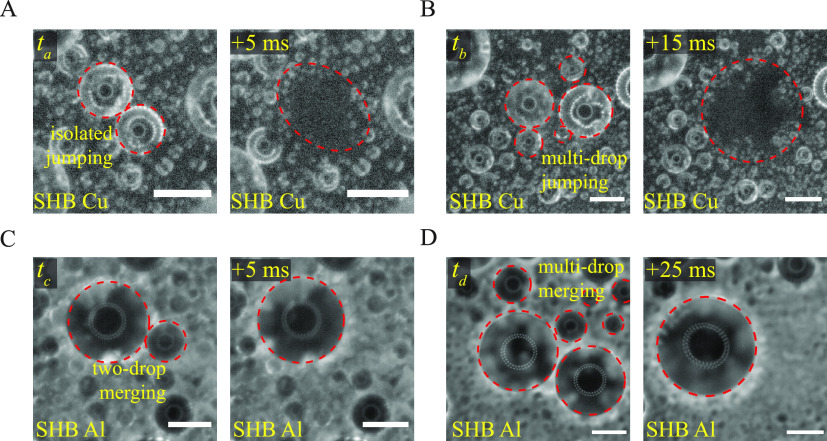
Microscale condensation on superhydrophobic surfaces. Coalescence-induced jumping (CIDJ) of (A) two-isolated drops and (B) multidrops on a SHB copper substrate. Drop merging of (C) two drops (D) multi drops during the coalescence of condensate drops on a SHB aluminum substrate. The environmental condition was *T*_env_ = 30°C and RH = 60%. Images were recorded using a high-speed camera at 200 fps using a zoom lens (Navitar-Zoom 6000). The resolution of the image was 1.06 μm/pixel. The scale bar is 100 μm for (a,b,c) and 200 μm for (d).

Figure 2 shows the zoomed-in view of the condensate droplets appearing at different instants of time on the superhydrophobic (SHB) surfaces of copper and aluminum, so as to discern the coalescence, sliding and jumping behaviors of millimetric and submillimetric condensate droplets. During the coalescence of microdrops on the SHB copper surface, the merged drop either jumped from the substrate or remained attached to the substrate, depending on whether the excess surface energy due to the coalescence could overcome the work of the solid–liquid adhesion or not.^41,42^Figure 2A shows the coalescence of two similar-sized drops, which caused out-of-plane jumping without disturbing any other neighboring drops. In contrast, the coalescence of asymmetric drops has been found to result in the spontaneous jumping of merged drops with an in-plane component of velocity. This in turn leads to further coalescence with neighboring drops, and results in multidrop jumping,^20^ as shown in Figure 2B. The interspace distance between the top microstructures of aluminum substrate is larger than the copper substrate (Figure 1), which increases the diffusion of water vapor to the crevices of the roughness features, allowing more condensate to form within the microfeatures.^40,42^ This causes a higher solid–liquid adhesion on the aluminum SHB surface due to pinning, and the condensate drops do not jump upon coalescence. In Figure 2C, during the coalescence of unequal-sized drops, the smaller droplets were completely depinned from their initial location and merged with the bigger droplet. When multiple drops were present in close proximity to the three phase contact line of the merging drops, the triggering of a single coalescence could also result in multidrop merging (see Figure 2D).

### Condensation Performance

**Figure 3:**
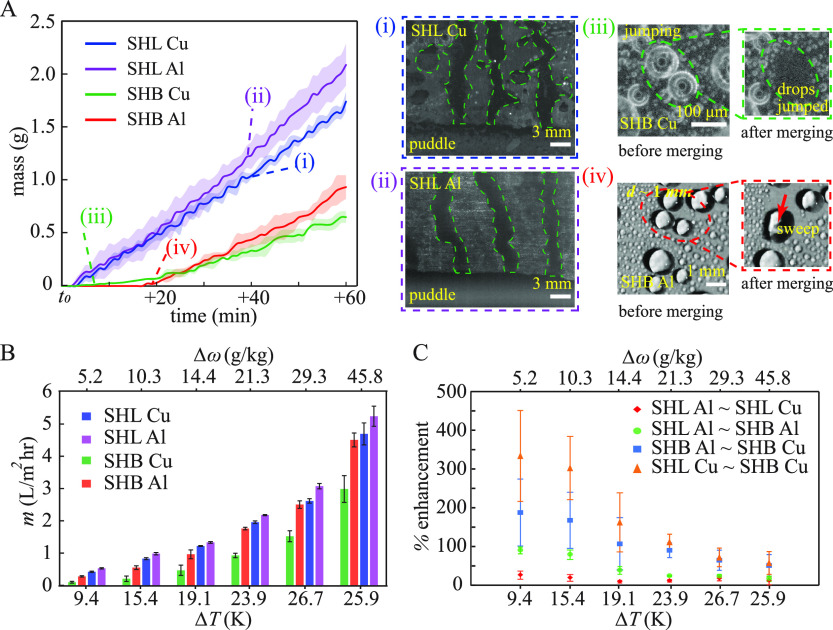
Water yield from different substrates. (A) The amount of water collected from the surfaces over time at a temperature of *T*_env_ = 30 °C, RH = 60%, and a surface temperature of *T*_s_ = 10 ± 0.5 °C. The measurement was recorded at a time interval of 1 minute using a precision balance of 10 mg accuracy. *t*_0_ is the time at which the surface temperature reaches the substrate temperature of *T*_s_. The shaded area denotes the uncertainty in the average mass of water collected from three repeated experiments. Inset images show the filmwise mode of condensation on a (i) copper superhydrophilic (SHL), (ii) aluminum superhydrophilic surface, (iii) coalescence-driven droplet jumping on superhydrophobic (SHB) copper surface, and (iv) condensate removal from superhydrophobic aluminum surface by sweeping. (B) Overall water collection rate of different surfaces under various environmental conditions is summarized in Table 2. (C) Percentage enhancement in condensation rate under different environmental conditions for the surfaces between SHL Al ∼ SHL Cu, SHL Al ∼ SHB Al, SHB Al ∼ SHB Cu, and SHL Cu ∼ SHB Cu.

Condensation experiments in the humid air environment were performed to compare the water collection rate from the fabricated surfaces at a degree of subcooling of 10–26 °C and a humidity ratio difference of 5–45 g/kg of dry air. The different experimental conditions chosen for this study are shown in Table 2. The condensation rate of the surfaces was evaluated by measuring the mass of the drained condensate from the surface using a precision balance at every experimental condition. Figure 3A depicts the amount of water collected in a container over time for different surfaces considered in this study. These experiments were performed at a temperature of *T*_env_ = 3 0°C, RH = 60%, and a surface temperature of *T*_s_ = 10 ± 0.5 °C. On superhydrophilic surfaces, the vapor nucleated easily due to the low energy barrier, and the condensate spread due to hemiwicking^43^ and formed a thin film that drained through the vertical surface due to gravity (Figure 3A (i) and (ii)). The drained condensate accumulated at the bottom of the surface as a puddle due to the pinning resistance at the bottom edge of the substrate. Therefrom, the condensate liquid was removed as the gravity force on the accumulated liquid eventually exceeded the pinning resistance. The first event of condensate drainage from superhydrophilic copper and aluminum substrates occurred almost the same time ∼ *t*_0_ + 3 minutes for the size of the condensate plate considered here. The trend of the water collection was linear with time for both superhydrophilic copper and aluminum surfaces, but the rate of condensation on the superhydrophilic aluminum surface was found to be higher than on the superhydrophilic copper surface (see the plot in Figure 3A).

**Table 2:** Experimental Conditions Considered in the Present Study Using Environmental Chamber^a^

environmental temperature *T*_env_, °C	relative humidity ϕ, %	Surface temperature *T*_s_, °C	dew point temperature *T*_dew_, °C	Degree of subcooling Δ*T*, °C	humidity ratio difference Δω, g/kg
20	75	6 ± 0.5	15.43	9.43	5.17
30	60	6 ± 0.5	21.39	15.39	10.26
30	75	6 ± 0.5	25.08	19.08	14.39
35	75	6 ± 0.5	29.89	23.89	21.25
40	75	8 ± 0.5	34.71	26.71	29.31
45	90	17 ± 1	42.97	25.97	45.82

a The chosen environmental conditions cover a wide range of degrees of subcooling and differences in humidity ratios.

Coalescence-induced droplet jumping was observed on superhydrophobic copper substrate^44,45^ which predominantly caused the droplet removal (Figure 3A(iii)). The coalescence-induced droplet jumping events have been identified as two-drop (Figure 2A) or multidrop jumping (Figure 2B). Furthermore, droplet sweeping events were also observed sporadically on the surface (Figure S1a). On the other hand, condensate drops from the superhydrophobic aluminum substrate were removed by gravity-assisted sweeping mode (Figure 3A(iv)). The jumping microdroplets can travel a longer distance from the substrate during their projectile motion. For the purpose of measurement of condensation rate, it is important to ensure that the condensate from all the surfaces reaches the condensate collection container without any mass loss inflicted by jumping droplets falling outside the container. The maximum possible velocity of the jumping droplet is in the order of ∼30 cm/s.^46^ In the current experiments, the height between the top edge of the substrate to the precision balance was around ∼15 cm. The horizontal distance traveled by the jumped droplet from the surface can be theoretically computed as  (equations of motion), and the corresponding value is ∼5 cm. We used a Petri dish of diameter 9 cm to collect the condensate from the substrate. Since the size of the Petri dish is larger than the maximum possible horizontal distance a jumping droplet can travel, we can safely assume that all the condensed droplets are collected, and there are no mass losses associated with jumping microdroplets. Coalescence of drops promptly ejected the condensate microdroplets from the superhydrophobic copper surface even before t_0_ minutes. The mass of such microdroplets was difficult to measure experimentally since they weighed less than the precision balance resolution (10 mg). However, with the accumulation of multiple such droplets in the collection container, measurement was possible from ∼*t*_0_ + 4 minutes onward, as shown in Figure 3A. Interestingly, the onset of water collection on the superhydrophobic aluminum substrate started at *t*_0_ + 19 minutes despite the surface having a very low contact angle hysteresis (below 5°). On the superhydrophobic aluminum surface, condensate was removed by gravity-assisted sweeping, and the drops exhibited a departure diameter of ∼1.4 ± 0.5 mm above which they were drained by gravity from the surface (Figure S1b). Although the drainage of condensate occurs on the superhydrophobic copper surface earlier than the superhydrophobic aluminum surface (Figure 3A), the water collection from the superhydrophobic aluminum surface surpasses that from the superhydrophobic copper substrate beyond *t*_0_ + 27 minutes. The water collection rate after the initial droplet drainage on the superhydrophobic aluminum surface is found to be higher than that on the superhydrophobic copper substrate because on the former, the drainage of a single droplet causes the additional removal of multiple droplets while sweeping.

A stable dropwise condensation mode was observed on superhydrophobic copper (Movie S1) and aluminum substrates (Movie S2) under all experimental conditions, as shown in Figure S2. In all cases, the superhydrophilic aluminum substrate had a higher water collection rate than all other surfaces, as shown in Figure 3B. The water collection rate increases with the humidity ratio difference and degree of subcooling.^47^ The condensation rate of the superhydrophobic aluminum surface was found to be higher than that of the superhydrophobic copper surface at all the experimental conditions. The coalescence-induced droplet jumping mechanism played a dominant role in the removal of the condensate from the superhydrophobic copper surface, while for the superhydrophobic aluminum surface, the condensate removal was primarily by gravity-assisted sweeping. This suggests that the vertically oriented surfaces exhibiting droplet jumping are less efficient for atmospheric humidity harvesting applications.^14^ Interestingly enough, the same phenomenon has been strongly attributed to enhancing the condensation heat transfer in pure vapor conditions.^45,48,49^ During pure vapor condensation, the nanoengineered superhydrophobic surface led to flooding at higher subcooling due to an uncontrolled nucleation rate.^21,45,50^ However, the event of flooding was not observed on the superhydrophobic surfaces during the humid air condensation experiments at higher subcooling (∼26 K) conditions even after 6 h. This indicates that the presence of NCG hampers the nucleation of fresh embryos on the subcooled substrate and helps in the prevention of flooding on superhydrophobic surfaces at higher subcooling conditions. Figure 3C compares the percentage enhancement of condensation rate between different surfaces under a wide range of environmental conditions. The superhydrophobic copper surface yielded 50–190% less condensate collection than the superhydrophobic aluminum surface since the surfaces exhibiting droplet jumping are less effective than the surfaces exhibiting sweeping mode of condensate removal in the atmospheric humid air environment.^14^ The filmwise mode on the superhydrophilic aluminum surface yielded 16–90% more condensate collection than the dropwise mode on the superhydrophobic aluminum surface with gravity-assisted drainage. The dropwise condensation on the superhydrophobic copper substrate yielded 57–333% lower condensation rate than the superhydrophilic copper surface. A reduction in condensation rate by 333% on superhydrophobic copper surfaces at low vapor content (Δω = 5.2 g/kg) indicates that the superhydrophobic surfaces, which can show droplet jumping during condensation, are the least effective surfaces for humidity harvesting.

### Condensation on Superhydrophilic Surfaces: Role of Rivulets

Another important observation from this study is that the condensation rate is higher on the superhydrophilic aluminum surface than the superhydrophilic copper surface, although both surfaces had a contact angle of almost 0°. The condensation rate of the superhydrophilic aluminum surface has shown an enhancement of 9–26% over the superhydrophilic copper surface. Importantly, at low vapor content (Δω = 5.2 g/kg), the superhydrophilic aluminum surface showed an enhancement of nearly 26%, which is essential for the humidity harvesting system because getting a higher condensation rate at a low humidity ratio situation is critical.^51^ The influence of surface textures of superhydrophobic surfaces on condensation was extensively investigated in the past^18−25^ because they exhibited enhanced performance compared to structured superhydrophobic surfaces^45^ under pure vapor conditions. However, such influences of superhydrophilic or hydrophilic surfaces were not reported. These results imply that micro/nano features on the superhydrophilic surface have a significant impact on condensation performance in humid air.

**Figure 4:**
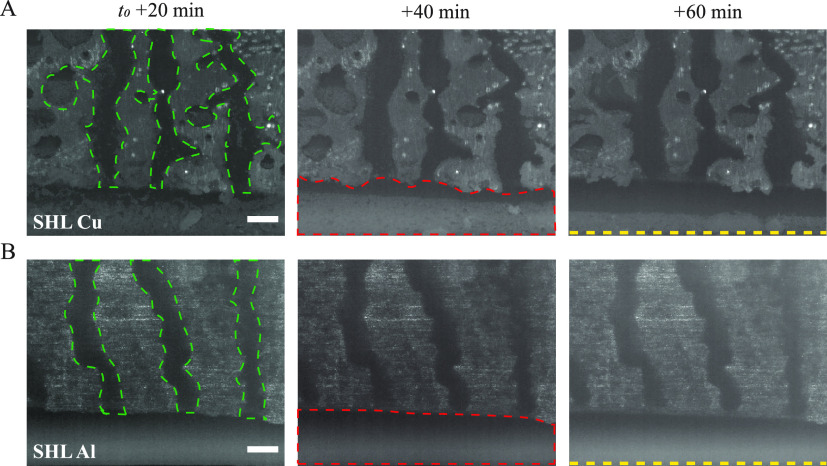
Rivulets on superhydrophilic surfaces. Time-lapse snapshots of condensation on superhydrophilic (SHL) (A) copper and (B) aluminum substrates at *T*_env_ = 27 °C, RH = 60%, *T*_dew_ = 18.6 °C, and *T*_s_ = 6 ± 0.5 °C. *t*_0_ is the time at which the surface temperature reaches the set value. Here, the green and red dashed regions represent the rivulet and puddle regimes, respectively. The Yellow dashed line represents the bottom edge of the substrate. For clear visualization of different regimes on superhydrophilic surfaces during condensation, we used IR imaging (FLIR-A655sc) to produce the above image. The scale bar is 3 mm.

At the initial stage of condensation on superhydrophilic vertical surfaces, the nucleated drops spread over the surface by coalescence and form the rivulet regime in which condensate is found to be drained downward by gravity. The condensate, drained through rivulets, accumulates at the bottom of the substrate as a puddle (marked as a red dashed line in Figure 4) due to the pinning force offered by the bottom edge. The puddle volume grows over time, and it eventually drains off the surface when gravity overcomes the pinning force at the bottom-edge. The size of the puddle reduces right after the drainage and then again increases with time until the next shedding happens. The nucleated condensate observed in the thin-film regime (the region outside of the puddle and rivulet regimes) spreads and merges with the rivulet or puddle. Gravity effects are negligible in the thin-film region. The rivulet regime has a thicker condensate film than the thin film regime. The shape of the rivulet depends on the surface topography and heterogeneity. The observed rivulet shape differed between copper and aluminum superhydrophilic substrates, and did not vary with time once they reached a steady-state (Figure 4).

The rivulets on the superhydrophilic aluminum substrate are mostly vertical channels (Movie S3), whereas the rivulets on the superhydrophilic copper surface consist of multiple branches that are inclined or horizontal and connected to the vertical channel (Movie S4). Rivulets form in the network of interstitial spacing between the microtextures and the condensate drain in the downward direction as the substrate placed in vertical orientation. The microtextures are closely packed on the SHL copper surface as compared to the SHL aluminum surface, and the height of the interstitial spacing is lower (see Figure 1A–D). The smaller and more closely packed microtextures on the SHL copper substrate inhibit flow in the vertical direction during condensate drainage, forming inclined branches of rivulets. The flow resistance is lower on the SHL aluminum substrate than SHL copper, since the interstitial spacing and height of the microtextures are greater on the SHL aluminum substrate. As a result, vertical-shaped rivulets emerge on the SHL aluminum surface. The condensate present in the vertical rivulet channel is smoothly drained due to gravity. The condensate accumulated in the rivulet branches is either stagnant or flowing with a lower velocity toward the central vertical channel due to the flow resistance offered from the accumulated condensate in the central channel. Thus, the overall drainage rate on the superhydrophilic copper substrate is reduced.

The above observations clearly elucidate that the condensation performance of a superhydrophilic surface under humid air conditions is significantly influenced by rivulet formation and the dynamics of the drainage mechanism through the rivulets. The spreading of condensate in the thin film region leads to the continuous transport of condensate from the thin film regions to the rivulet regions. This may have caused, for the range of degree of subcooling and humidity ratio difference investigated here, the peak of the rough structures on the SHL copper and aluminum substrates to remain dry and protruded above the thin film.^32,35,52^ These regions act as the sites for a high rate of heterogeneous nucleation despite having the substrate exhibiting an overall thin-filmwise condensation. As a result, at steady state, the rate of condensate formation in the thin-film regime is higher than that in the rivulet regime. Overall, it is clear that an optimal superhydrophilic surface with only vertically shaped rivulet channels can improve the condensation rate for humidity harvesting.

Notably, under all experimental conditions considered in this work, condensation mass transfer in the filmwise mode of condensation on the superhydrophilic surface was greater than that in the dropwise mode of condensation on the superhydrophobic surface (Figure 3B). This observation contradicts the general perception of condensation under pure steam conditions.^15−17^ Although multiple research works summarized in Table 1 showed observations similar to our foregoing experimental results, the reason for this observation was not explained in any of these studies. A detailed comparison between condensation phenomena in the pure vapor and NCG environments is necessary and presented next.

### Influence of Noncondensable Gases

To understand the influence of NCGs during condensation, a theoretical analysis of a single condensing drop^36,37^ and a condensate film^53^ was carried out for a wide range of NCG concentrations. This section compares the nucleation performance of different surfaces and the individual contribution of temperature drop due to different thermal resistances at a moderate and exemplar subcooling of 10 K.

The nucleation rate of a substrate is defined as the probability of the liquid embryo that is continuing to grow on a nucleation site without evaporating during vapor–liquid phase conversion. The heterogeneous nucleation rate of a substrate per unit area (*dN*/*dt*, in m^–2^s^–1^) can be expressed as,^6^
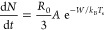
1where *R*_0_ is the radius of the water molecule, *R*_0_ ≈ 1.375 × 10^–10^ m, *A* is the Arrhenius prefactor, *W* is the total energy required for the formation of a liquid nuclei on a substrate with water drop contact angle of θ, *k*_B_ is the Boltzmann constant, and *T*_s_ is the substrate temperature (refer to Supplementary section S1 for more details).

**Figure 5:**
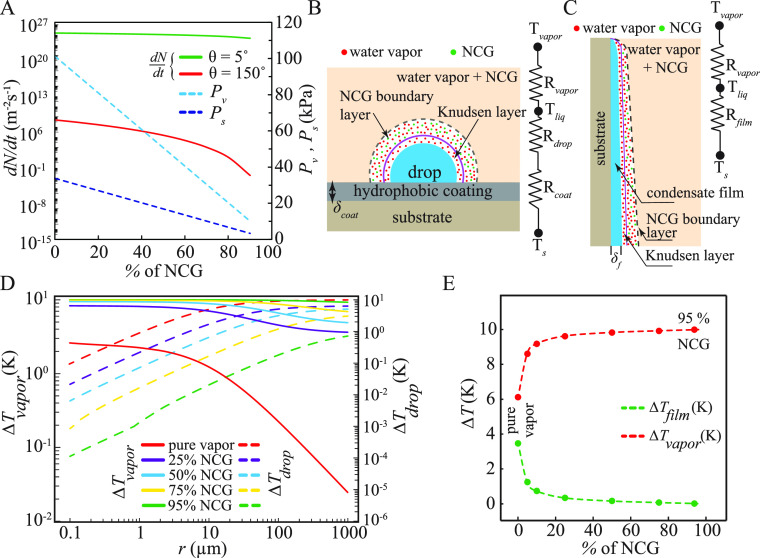
Nucleation rate and temperature drops in different modes of condensation. (A) The influence of NCG concentration and surface wettability on heterogeneous nucleation rate for a fixed supersaturation ratio, *S* = *P*_v_/*P*_s_ = 3. Schematic of heat transfer model with thermal resistance network across (B) a single droplet during dropwise condensation and (C) a film during filmwise condensation in the presence of NCGs. The influence of NCG concentration on temperature drop across the liquid (film or droplet) due to conduction (Δ*T*_drop/film_) and temperature drop across the vapor region (Δ*T*_vapor_) during (D) dropwise mode and (E) filmwise mode at a subcooling of 10 K. The thermal conductivity (*k*_coat_) and the thickness of the hydrophobic layer coating (δ_coat_) are approximated as 0.2 W/mK and 1 μm, respectively (data from ref (37)). δ_f_ is the average condensate film thickness, and it is approximated as 25 μm (data from ref (54)). Refer to Supplementary section S1 for more details.

The vapor pressure is significantly reduced with the increase in NCG concentration (Figure 5A). It also reduces the heterogeneous nucleation rate.^6^ The accumulation of NCGs near the subcooled surface can further reduce water vapor diffusion from the bulk region to the vapor–liquid interface and, thus, hinder the nucleation rate. Figure 5A compares the influence of NCG concentration on nucleation rate for surfaces with extreme wettability (such as superhydrophilic and superhydrophobic) at a supersaturation ratio (*S*) of 3. The supersaturation ratio is defined as the ratio of partial pressure of the water vapor (*P*_v_) to the saturation pressure corresponding to the substrate temperature *T*_s_ (*P*_s_). As can be seen from Figure 5A, the nucleation rate decreases with NCG concentration; at 90% NCG concentration, the nucleation rate of the superhydrophilic surface reaches a value that is 1 order of magnitude lower than that for the pure vapor condition. On the other hand, the nucleation rate of a superhydrophobic surface reaches a value that is 11 orders of magnitude lower than that of the pure vapor condition, as shown in Figure 5A. As a result, nucleation on superhydrophobic surfaces occurs much more slowly from humid air than from pure vapor. The interplay between the phase transition of vapor molecules and vapor diffusion from the bulk fluid makes the nucleation phenomena complex in the presence of NCG. The probability of activating a nucleation site in the NCG environment depends significantly on the localized vapor content and vapor diffusion rate across the NCG diffusion layer.

Figure 5B and C shows a schematic representation of the thermal resistance network model in the presence of NCG across a single droplet for the dropwise condensation and across the film for the filmwise condensation. The schematics consist of three regions: the hydrophobic coating region, the condensed drop/film region, and the vapor region. The vapor region consists of an interfacial region called the Knudsen layer, the diffusion layer, and the bulk vapor-NCG mixture region. In the Knudsen layer, the kinetic theory of gases governs the transfer of vapor molecules to the liquid drop interface. The region outside the Knudsen layer is known as the diffusion layer, and during condensation, a concentration gradient of vapor exists in this region. The thickness of the diffusion layer depends both on the hydrodynamic parameter (the free stream velocity for a forced flow and the Grashof number in the case of a thermogravitational flow) and the thermophysical properties (e.g., thermal and mass diffusivities). Thermo-gravitational Grashof number is defined as
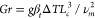
2where *g* is the acceleration due to gravity, β_t_ is the coefficient of thermal expansion, *L*_c_ is the characteristic length scale, and ν_m_ is the kinematic viscosity. For the present case, considering the length of the vertically mounted plate (80 mm) to be the characteristic length scale (*L*_c_), the magnitude of the thermogravitational Grashof number is ∼10^5^–10^6^. The diffusion layer thickness is expressed as
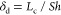
3where *Sh* is the pertinent Sherwood number and can be calculated from the correlation applicable for the vertical surface under natural convection,
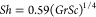
4where *Sc* is the Schmidt number, defined as the ratio of the kinematic viscosity (ν_m_) of the humid air to the mass diffusion coefficient (*D*_m_) of the vapor molecules in the humid air. A change in the air temperature and the bulk vapor concentration alters the average mass and thermal diffusivities, thereby altering the diffusion layer thickness.^55^ The thickness of the diffusion layer increases as the water vapor concentration of the mixture decreases. Outside of the Knudsen outer interface, transport is governed by combined mass diffusion and energy conservation laws. For condensation under a pure steam scenario, the vapor in the bulk region remains in a state of thermal equilibrium, and the interface temperature of the drop is always maintained at the saturation temperature corresponding to the vapor pressure. However, the presence of NCGs add heat and mass transfer resistances, thus rendering the actual prediction of interface temperature difficult. It can only be predicted by solving coupled mass and energy conservation equations.^10,55,56^ The total temperature difference (Δ*T*) between the cold surface and the bulk vapor–NCG mixture is expressed as follows:
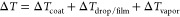
5where Δ*T*_coat_, Δ*T*_drop/film_, Δ*T*_vapor_ are the temperature drops across the promoter layer coating, liquid drop/film due to conduction, and vapor region, respectively. In the present analysis of a fixed subcooling of Δ*T*, the Δ*T*_vapor_ is calculated by difference, following eq 5. It is important to note that the Δ*T*_vapor_ includes the temperature drop due to curvature resistance (Δ*T*_c_, see eq S13) and the interfacial temperature drop (Δ*T*_int_, see eq S14). The estimation of each temperature drop during dropwise and filmwise modes of condensation is calculated from the numerical iteration of analytical expressions for the case of atmospheric water vapor or an existing correlation for the case of pure vapor (detailed discussions can be found in Supplementary section S1).

The individual contribution of temperature drop over the condensate drop and vapor region for a drop of radius 100 nm to 1 mm during dropwise mode condensation of pure vapor and the vapor–NCG mixture at a moderate subcooling of 10 K is shown in Figure 5D. In the dropwise mode of condensation, the temperature drop across the vapor region is significantly higher for the droplets of radius below 1 μm irrespective of the NCG concentration. This is because the curvature component (Δ*T*_c_) offers greater thermal resistance for smaller droplets. The contribution of these thermal resistances to the overall temperature drop decreases as the drop size increases. In the case of pure vapor condensation, the temperature drop across the droplet is significant except for the case of smaller droplets of radius below 0.1 μm and it reaches above 90% of the total subcooling for droplets with a radius greater than 200 μm. The temperature drop across the drop due to conduction decreases as NCG concentration increases. With 95% NCG (for example, atmospheric humid air), the temperature drop within the condensate droplet is entirely negligible for any droplet size (Figure 5D), and the magnitude of the temperature drop across the coating is negligible (Figure S3), even at a coating thickness of 10 μm. For filmwise condensation, variation in the temperature drop across the film and vapor regions with different NCG concentrations is shown in Figure 5E. Both the temperature drops (across the film and vapor region) are significant, and they occur in the same order as for pure vapor condensation. However, the temperature drop across the film decreases with NCG concentration and becomes negligible at concentration greater than 50%. Moreover, the temperature drop occurs primarily in the vapor region.

Therefore, regardless of the mode of condensation, the heat transfer performance during humid air condensation is primarily controlled by the rate of diffusion of the vapor molecules through the NCG diffusion layer, and the thermal resistance offered by the condensate drop or the coating is insignificant. The heat transfer performance during the filmwise mode is poor in a pure vapor environment due to the significant thermal resistance across the condensate film. In humid air condensation, however, a similar conclusion is incorrect because the thermal resistance across the film/drop is negligible and the interface temperature of the film/drop is close to the substrate temperature.^10^ In addition to this, the nucleation rate of the superhydrophilic surface is 27 orders of magnitude higher than the superhydrophobic surface for a supersaturation ratio of 3 (Figure 5A). As a result, rejuvenation of heterogeneous nucleation sites after the drop removal is energetically expensive for superhydrophobic surfaces in humid air conditions. Based on these understandings and observations from the experiments, we can interpret that superhydrophilic surfaces can show better condensation heat transfer performance over the superhydrophobic surface in the humid air environment with more than 95% NCGs, and they can outperform in atmospheric humidity harvesting applications.

In practical atmospheric water harvesting systems, the condensing surfaces are exposed to ambient air and tend to absorb airborne molecules like volatile organic compounds (VOCs) and secondary organic aerosols (SOAs).^57^ Long-term operations may result in the accumulation of water-soluble airborne molecules on the micro/nano structures despite the substrate being covered with condensate film. This can only alter the chemical characteristics of the interface, preserving the physical structure unchanged and leading to increase in the water drop contact angle over the substrate. A rigorous analysis is required to evaluate the water collection efficiency and long-term performance of fabricated nanoengineered surfaces upon VOCs adsorption from ambient air. In addition, other durable superhydrophilic fabrication techniques, like as anodization, can be examined for long-term performance during condensation of atmospheric water vapor, as the oxide layer produced by anodization is less reactive to airborne molecules.^58,59^

In this study, the condensing surface was cooled below the dew point with the help of a Peltier module. Similarly, exposing the substrate to a cold refrigerant fluid can also result in the cooling of the substrate. These methods are active cooling techniques, and a large amount of electricity needs to be utilized for atmospheric water harvesting that uses these methods of cooling. On the other hand, radiative cooling is a promising technology for atmospheric water harvesting as it uses the natural cooling effect of the atmosphere to condense water from the ambient air and does not require any additional energy inputs.^5,6^ Currently, the radiative cooling technique for atmospheric water harvesting is mainly in the small-scale prototype stage and less efficient during the daytime.^4^ These technologies can be scaled by improving the radiative cooling efficiency of the new materials, which can provide uninterrupted cooling regardless of sunlight conditions.^7,8^ The efficiency of the atmospheric water harvesting devices that use radiative cooling technology is directly proportional to the effective emissivity of the condensing surface.^60^ During sustained dropwise condensation mode, nearly 20–50% of the surface is covered by condensate droplets, and the remaining area remains as dry.^48^ Hence, during the dropwise condensation mode, the effective emissivity will be in the intermediate range of the water and substrate emissivity. In the filmwise condensation mode, the effective emissivity became equal to the high water emissivity (∼0.98).^60^ Higher emissivity during filmwise mode may provide an additional benefit during water harvesting that uses radiative cooling. Further, experimental testing of radiative cooling-based atmospheric water harvesters under different climatic conditions using other surfaces in different regions is required to determine their effectiveness and potential for widespread adoption.

## Conclusions

The major challenge in humidity harvesting devices is obtaining the required amount of water yield from the atmospheric humid air on a daily basis. The air temperature and relative humidity vary throughout the day, and the water yield from the surface can be drastically reduced, particularly when the humidity ratio is low. As a result, increasing water yield under low humidity conditions is critical for obtaining the desired water yield from the humidity harvesting system. The superior condensation rates on superhydrophilic surfaces compared to the superhydrophobic surface at low humidity ratios is appealing for humidity harvesting systems. From other aspects also, such as the cost associated with surface fabrication, scalability of the surface fabrication technique, and chemical contamination possibilities of condensate with hydrophobic coating material, superhydrophilic surface appears to be more suitable than the superhydrophobic surfaces for humidity harvesting.

In summary, this study investigated the condensation performance of vertically oriented superhydrophilic and superhydrophobic surfaces in a wide range of humid air environments. Interestingly, at a vapor concentration of below 5%, we found that the filmwise mode of condensation on a superhydrophilic surface shows superior water collection compared to the dropwise mode of condensation observed on a superhydrophobic surface. Theoretical analyses indicate that under humid air conditions, the conduction thermal resistance across the condensate liquid or the surface coatings is negligible, and the condensation performance is driven by diffusion and drainage rates. Hence, film formation does not significantly affect the overall condensation performance, unlike what usually happens in the pure steam environment.^15,16^ Besides, the very high nucleation rate of a superhydrophilic surface at the peaks of the microfeatures in the thin film regime can substantially increase the condensation rate. However, a wettability-patterned surface with an optimum nucleation rate and drainage rate, a liquid-infused substrate, or soft substrates may produce a higher water yield than a homogeneous superhydrophilic surface. In effect, our study provides a new mechanistic understanding of why the filmwise mode of condensation is better for humidity harvesting, as well as an insight into exploiting the new physics of rivulets to maximize this effectiveness. The key findings of this study can usher in a new pathway to the development of efficiently engineered surfaces for humidity harvesting systems.

## Materials and Methods

### Surface Preparation and Characterization

Aluminum alloy (Al-6061) and copper (99.9% purity) plates of size 80 mm × 40 mm × 2 mm were used as the substrate for the condensation experiments. First, the substrates were mechanically polished with sand paper of grade 220 to 2000 sequentially. Then the polished substrate was washed with DI water before being ultrasonically cleaned for 10 min in a solution of DI water, ethanol, and acetone. The clean copper substrate was kept in a 3 M HNO_3_ (Merck, Emplura grade) solution for 10 min to remove the native oxides, and further rinsed with DI water and dried with nitrogen gas. Thereafter, the substrate was oxidized in an aqueous solution of 2 M NaOH (Sigma-Aldrich, ACS reagent grade pellets) and 0.1M K_2_S_2_O_8_ (Sigma-Aldrich, ACS reagent grade) for 1 h,^19^ yielding a superhydrophilic surfaces. Next, the substrate was rinsed with DI water, and dried with nitrogen gas, and kept in the hot air oven for 30 min at a temperature of 150 °C. To achieve superhydrophobicity, the oxidized copper substrate was dip-coated for 4 h in an ethanol solution with 0.5% perfluorooctyltriethoxysilane (Sigma-Aldrich) and dried in the hot air oven at 150 °C for 1 h.

The aluminum substrate was microtextured by dipping it in a 3 M solution of HCl (Merck, Emplura grade) for 5 min and then rinsing it with DI water. The chemically etched aluminum substrate was further immersed in a hot DI water bath at a temperature of 100 °C for 30 min to generate the nanostructures above the microstructures.^61^ The aluminum substrate became superhydrophilic after these fabrication steps. Next, the substrate was functionalized with 0.5% perfluorooctyltriethoxysilane in ethanol solution for 4 h and then dried in the laboratory environment for 12 h for achieving superhydrophobicity.

A high-resolution scanning electron microscope (Inspect F-FEI) was used to examine the surface morphology. The roughness of the modified substrate was measured using a noncontact type profilometer (NT1100-Wyco). The static contact angle of water on the substrate was measured with a goniometer (Holmarc) by placing a sessile drop of volume 5 μL using a micropipette. The dynamic contact angles were determined by injecting/drawing DI water into/from the sessile drop with a syringe pump at a creeping flow rate of 0.1 μL/ s.^62^ Each reported contact angle comprises of the average of six measurements taken at various locations on the substrate.

### Experimental Procedures

The condensation experiments for a vertically oriented surface were performed in an environmental chamber (PR2J-Espec) of size 500 × 750 × 600 mm^3^ at different environmental conditions as shown in Figure S4. The fluctuations in air temperature and relative humidity were ±0.5°C and ±3%, respectively. The substrates under test were attached to a Peltier (CP061HT-Tetech) element with thermal conductive tape, and the remaining area of the Peltier element was covered with nitrile foam to avoid condensation on the Peltier cooling plate. The cooling capacity of the Peltier module at different experimental conditions during this study ranged from 25–50 W. The temperature of the Peltier element was controlled using a PID controller. Four holes of size 0.8 mm were drilled on the lateral side of the substrate for the insertion of K-type thermocouples (Omega, 0.13 mm bead diameter) for surface temperature measurement. The temperature from the thermocouples was logged onto a data acquisition system (DAQ970A-Keysight) at every minute. The condensing substrate was placed at the center of the environmental chamber to avoid the boundary wall effects. The concentration boundary layer can be disturbed by the air circulation near the condensing surface, which can change the rate of vapor diffusion and condensate droplet removal. A thermal anemometer (Testo 445) probe was used to measure the velocity of the humid air inside the environmental chamber. Numerous velocity measurements at various climatic conditions were made to characterize the air velocity at a plane 8 mm away from the surface. The probe was placed at the top, middle, and bottom of the surface to measure velocity. The velocity of the bulk humid air near the surface varies from 0.1 to 1.1 m/s depending on the environmental conditions and the spatial location. The spatiotemporal RMS velocity was 0.54 ± 0.38 m/s. This study did not examine the air convection effects during the condensation process. The condensation experiments were performed for 2–3 h, and the condensed water was collected in a Petri dish with a diameter of 9 cm. This ensured that the condensate from all the surfaces reached the container while jumping or sweeping. Nonetheless, some mass loss may occur due to evaporation because the collected condensate in the Petri dish is exposed to the unsaturated humid air surrounding it. The current research aims to produce an efficient atmospheric water harvesting generator, and evaporation losses cannot be totally eliminated because the collection system is an open system. Nevertheless, evaporation losses can be reduced by designing an improved collection system.^51^ The mass of the water from the Petri dish was measured in a precision balance (SPX622-Ohaus) with a precision of 10 mg. At least three experiments were performed to calculate the average condensation rate on a surface. The condensation images were captured with a DSLR camera (D750-Nikon) and a 105 mm macro lens at 30 fps. An LED spotlight was used to illuminate the imaging area (CW8-Moritex).

## Data Availability

The data that support the findings of this study are available from the corresponding authors upon request.

## Supporting Information Available

Macroscale condensation observation on superhydrophobic copper substrate for a duration of 15 min; the video is recorded using a digital camera at 1 fps, and the playback speed is 10× (MP4)
Macroscale condensation observation on superhydrophobic aluminum substrate for a duration of 15 min; the video is recorded using a digital camera at 1 fps, and the playback speed is 10× (MP4)
Illustration of condensate growth, rivulet formation, and drainage on superhydrophilic aluminum substrate for a duration of 1 h; the video is recorded using an IR camera at 1 fps, and the playback speed is 30× (MP4)
Illustration of condensate growth, rivulet formation, and drainage on superhydrophilic copper substrate for a duration of 1 h; the video is recorded using an IR camera at 1 fps, and the playback speed is 30× (MP4)
Influence of noncondensable gases under different modes of condensation through analytical modeling; condensate drainage mechanism on superhydrophobic surfaces; time-lapse snapshots of dropwise condensation on superhydrophobic surfaces; temperature drop across the hydrophobic coating of different thickness; experimental setup for macroscale condensation studies (PDF)
