# Supplementary material for: Preferred Mode of Atmospheric Water Vapor Condensation on Nanoengineered Surfaces: Dropwise or Filmwise?
Source: Langmuir. 2023 Apr 4;39(15):5396–407. doi: 10.1021/acs.langmuir.3c00022 (PMC10116598; doi:10.1021/acs.langmuir.3c00022)
Supplement: Supplementary file 5 — la3c00022_si_005.pdf [file la3c00022_si_005.pdf]

Supplementary Information

Preferred mode of atmospheric water vapor condensation on nanoengineered surfaces: Dropwise or filmwise?

Tibin M Thomas,[†] Pallab Sinha Mahapatra,[*,†] Ranjan Ganguly,[‡] and Manish K. Tiwari[*,¶,§]

†*Department of Mechanical Engineering, Indian Institute of Technology Madras, Chennai 600036, India*

‡*Department of Power Engineering, Jadavpur University, Kolkata 700106, India*

¶*Nanoengineered Systems Laboratory, UCL, London WC1E 7JE, UK*

§*Wellcome/EPSRC Centre for Interventional and Surgical Sciences, UCL, London W1W 7TS, UK*

E-mail: pallab@iitm.ac.in; m.tiwari@ucl.ac.uk

Section S1: Influence of non-condensable gases during condensation

Nucleation

Condensation initiates with nucleation, i.e., the formation of thermodynamically stable liquid embryos on a substrate at specific sites depending on the surface roughness, wettability of a

substrate, supersaturation ratio, etc. The heterogeneous nucleation rate of a substrate per unit area having a water drop contact angle of θ, is given by[1]

$$\frac{dN}{dt} = \frac{R_0}{3} A e^{-\frac{W}{k_B T_s}} \tag{S1}$$

where R_0 is the radius of the water molecule, $R_0 \approx 1.375 \times 10^{-10}\ m$, A is the Arrhenius prefactor, W is the total energy required for the formation of a liquid nuclei on a substrate and T_s is the substrate temperature. The expression of W is as follows,

$$W = \frac{\beta_n^3}{27\alpha_n^2}(2 - 3cos\theta + cos\theta^3) \tag{S2}$$

where θ is the contact angle, $\alpha_n = k_B T_s ln(S)$ and $\beta_n = 4\pi\sigma_{lv}\big(\frac{3v_m}{4\pi}\big)^{2/3}$.

Here S is called as supersaturation ratio and it is the ratio of partial pressure of the vapor (P_v) to the saturated vapor pressure (P_s) corresponds to substrate temperature T_s (P_s), σ_{lv} is the surface tension of water, v_m is the volume occupied by a liquid molecule. The magnitude of v_m for water is $v_m \approx 3 \times 10^{-29}\ m^3$. The partial pressure of the water vapor (P_v) at different NCG ratios was calculated by multiplying the total pressure (atmospheric pressure) with the percentage fraction of water vapor present in the water vapor-air mixture.

In this study, the silane functionalization procedure was applied to make a superhydrophobic hierarchical interface. This process results in the formation of a thin layer of self-assembled $CF_3(CF_2)_5(CH_2)_2SiO-$ chains at the solid-liquid interface, followed by vapor nucleation on the coated monolayer. In a previous work, molecular dynamics simulations revealed that the droplets on this monolayer during vapor-liquid conversion were hemispherical.[2] Even at the nanoscale level, the macroscopic contact angle measured on the superhydrophobic surface was retained. Therefore the magnitude of θ in the Eq. S2 can be approximated as the macroscopic contact angle. The magnitude of the Arrhenius prefactor is evaluated as

$$A = c_0 Z f^* \tag{S3}$$

Here, c_0 is the concentration of the available nucleation sites and is expressed as

$$c_0 = \frac{P_m}{k_B T_s} \tag{S4}$$

where P_m is the total pressure of the humid air. The term Z is called as the Zeldovich parameter and expressed as

$$Z = \frac{3\alpha_n^2}{4\beta_n^2}\left(\frac{\beta_n}{\pi k_B T_s}\right)^{1/2} \tag{S5}$$

f^* is called as attachment frequency and mathematically expressed as

$$f^* = (48\pi^2 v_m)^{1/3}\alpha_m S x_s D_{vap} n^{*1/3} \tag{S6}$$

where D_{vap} is the diffusion coefficient of water vapor molecules in air and calculated from the Chapman-Enskog theory,[3]

$$D_{vap} = 2.11 \times 10^{-5}\left(\frac{T_v}{273.15}\right)^{1.94}\left(\frac{1}{P_m}\right) \tag{S7}$$

where T_v is the temperature of the humid air. α_m is the monomer sticking coefficient and approximated as 1,[1] x_s is the saturated water molecule concentration in air and calculated from ideal gas equation

$$x_s = \frac{P_s}{k_B T_s} \tag{S8}$$

n^* is the number of molecules occupied by the nuclei having a critical radius of r_{cr} and given by,

$$n^* = \left(\frac{2\beta_n}{3\alpha_n}\right)^{2/3} \tag{S9}$$

Temperature drop

The major driving parameters for humid air condensation are degree of subcooling (ΔT) and humidity ratio difference ($\Delta\omega$).[4] A theoretical analysis was performed for a wide range of

non-condensable gas (NCG) concentrations in order to better understand the role of NCG during condensation. This section compares the individual contribution of temperature drop due to different thermal resistances at a moderate subcooling of 10 K for dropwise and filmwise mode of condensation.

Dropwise condensation

Figure 5B shows a schematic representation of the thermal resistance network model of a single droplet for dropwise condensation in the presence of NCG. The schematic identifies three regions pertinent to the thermal resistance: the promoter layer coating region, the condensed drop/liquid region, and the vapor region. The vapor region includes an interfacial region called Knudsen layer and a surrounded diffusion layer region.

The total temperature difference (ΔT) between the cold surface and bulk vapor region for a single droplet can be expressed as,

$$\Delta T = \Delta T_{coat} + \Delta T_{drop} + \Delta T_{vapor} \tag{S10}$$

where ΔT_{coat} is the temperature drop across the promoter layer coating due to conduction and mathematically expressed as,

$$\Delta T_{coat} = \frac{q_d \delta_{coat}}{k_{coat} \pi r_d^2 sin^2\theta} \tag{S11}$$

where q_d is the heat transfer through a drop of radius r_d, δ_{coat} is the thickness of the coating, k_{coat} is the thermal conductivity of the coating, and θ is the equilibrium contact angle of a sessile water droplet on the coated surface. The temperature drop across the condensate drop (ΔT_{drop}) due to conduction is given by,[5]

$$\Delta T_{drop} = \frac{q_d \theta}{4\pi r_d k_l sin\theta} \tag{S12}$$

where k_l is the thermal conductivity of the condensate liquid. The temperature difference in the vapor region (ΔT_{vapor}) is calculated based on the bulk vapor conditions.

In the case of pure vapor condensation, the temperature at the outer interface of the Knudsen layer (T_i) maintained as saturation temperature (T_{sat}) corresponding to the ambient pressure and vapor region keeps on an equilibrium state. ΔT_{vapor} is defined as the sum of two temperature drops such as ΔT_c and ΔT_{int}. Wherein, the temperature drop due to curvature resistance (ΔT_c) is expressed as,

$$\Delta T_c = \frac{2T_{sat}\sigma_{lv}}{h_{lv} r_d \rho_l} = \frac{r_{cr}}{r_d}\Delta T \tag{S13}$$

where h_{lv} is the latent heat for vapor-liquid phase change, ρ_l is the density of the condensate liquid and r_{cr} is the critical radius for nucleation. The interfacial temperature drop (ΔT_{int}) is expressed as,

$$\Delta T_{int} = \frac{q_d}{h_{int} 2\pi r_d^2 (1 - cos\theta)} \tag{S14}$$

In the case of the condensation of pure vapor h_{int} is calculated from,[6]

$$h_{int} = \frac{2\epsilon}{2-\epsilon}\frac{1}{\sqrt{2\pi R_v T_{sat}}}\frac{h_{lv}^2}{v_v T_{sat}}\left(1 - \frac{p_v v_v}{2h_{lv}}\right) \tag{S15}$$

where ϵ is the accommodation co-efficient, R_v is the gas constant for the water vapor, p_v saturated vapor pressure corresponding to T_{sat}, and v_v is the specific volume. The magnitude of ϵ is dependent on the condensation conditions and is approximated as 0.02 for pure vapor conditions.[7] The heat transfer rate across a single droplet of radius r_d during pure vapor condensation can be calculated using Eq. S10 after substituting Eq. S11 - S14,

$$(q_d)_{pure\,vapor} = \frac{\Delta T \pi r^2 (1 - \frac{r_{cr}}{r_d})}{\left[\frac{\delta_{coat}}{k_{coat} sin^2\theta} + \frac{r_d \theta}{4k_l sin\theta} + \frac{1}{2h_{int}(1-cos\theta)}\right]} \tag{S16}$$

In the case of condensation in the presence of NCG, a diffusion layer forms outside the

interfacial region and a temperature gradient of vapor exists in this region during condensation. Therefore, interfacial temperature outside the Knudsen layer can only be predicted from the coupled solution of mass diffusion and energy conservation laws. Hence the heat transfer rate, the interface temperature between the coating and drop (T_{coat}), the surface temperature of the drop (T_d), and the Knudsen region outer layer temperature (T_i) for each condensing drops are calculated numerically by simultaneous solution of mass and energy conservation laws.[8,9]

In the Knudsen layer, the kinetic theory of gases governs the transfer of vapor molecules to the liquid drop interface. The effect of molecular collision in the Knudsen layer is negligible, and the thickness of the Knudsen layer is in the order of mean free path (λ_m) of the vapor-NCG mixture which can be mathematically expressed as,[10]

$$\lambda_m = \frac{2\mu_m\sqrt{R_m T_m}}{P_m} \tag{S17}$$

where μ_m, R_m, T_m, P_m are the dynamic viscosity, gas constant, temperature and pressure of the vapor-NCG mixture respectively. The outer radius of the Knudsen layer (r_i) is expressed as

$$r_i = r_d + \beta_k \lambda_m \tag{S18}$$

where r_d is the condensed drop radius, β_k is a constant and the best fit value for β_k is 0.75.[11]

The heat transfer rate across the droplet and coating region is governed by the Fourier law of heat conduction. The mass transfer rate through the coating and the droplet region is given by,

$$\dot{m} = \frac{T_d - T_s}{h_{lv}\left[\frac{\delta_{coat}}{k_{coat} sin^2\theta} + \frac{r_d\theta}{4k_l sin\theta}\right]} \tag{S19}$$

The mass transfer rate across the Knudsen layer region is calculated from the kinetic theory of gases,[9]

$$\dot{m} = \frac{4\pi r_d^2 r_i^2(1-cos\theta)}{2r_i^2 - \alpha_c r_d^2}\left(\frac{\alpha_c\rho_{v,i}R_v T_i}{\sqrt{2\pi R_v T_i}} - \frac{\alpha_c\rho_{v,d}R_v T_d}{\sqrt{2\pi R_v T_d}}\right) \tag{S20}$$

where $\rho_{v,i}$, $\rho_{v,d}$ are the water vapor density at the Knudsen interface and liquid drop surface, R_v is the gas constant of the water vapor. The magnitude of $\rho_{v,i}$, $\rho_{v,d}$ are calculated from the ideal gas equation as,

$$\rho_{v,i} = \frac{P_i}{R_v T_i} \tag{S21}$$

$$\rho_{v,d} = \frac{P_d}{R_v T_d} \tag{S22}$$

where the vapor pressure at the Knudsen interface is given by[3]

$$P_i = 611.2\, exp\Big[6816\Big(\frac{1}{273.15} - \frac{1}{T_i}\Big) + 5.1309\, ln\Big(\frac{273.15}{T_i}\Big)\Big] \tag{S23}$$

and the vapor pressure at the curved interface of the droplet surface is derived from the Kelvin equation as follows,[8]

$$P_d = exp\left(\frac{2\sigma_{lv}}{\rho_l R_v T_d r_d}\right)\left[611.2\, exp\Big[6816\Big(\frac{1}{273.15} - \frac{1}{T_d}\Big) + 5.1309\, ln\Big(\frac{273.15}{T_d}\Big)\Big]\right] \tag{S24}$$

In Eq. S20, the variable α_c is condensation coefficient and the magnitude of α_c vary with the vapor conditions.[9] Consequently, this fitting parameter must be computed separately based on the NCG concentration present in the water vapor-air mixtures. The magnitude of α_c at higher NCG condition ($\sim$ 97%) is approximated by validating the numerical model with *Zheng et al.* experiments[9] and the lower NCG condition ($\sim$ 5%) is approximated by validating the numerical model with *Wen et al.* experiments.[12] The value of α_c is found to be 0.0003 and 0.0075 for the NCG mass fraction of $\sim$97% and $\sim$5% respectively. The α_c value for other NCG fractions is approximated from the exponential fitting curve between the computed α_c magnitudes for the NCG mass fraction of 97% and 5%. The approximated exponential fitting formula for the condensation coefficient was α_c=0.0089$e^{-3.514\omega_a}$, wherein ω_a is the percentage of air content in the mixture.

The mass flux across the diffusion layer is calculated based on the Fick's law of diffusion

given by,

$$\dot{m} = 2\pi r_i(1 - cos\theta)D_m(\rho_{v,m} - \rho_{v,i}) \tag{S25}$$

where $\rho_{v,m}$ is the density of water vapor outside the diffusion layer based on the vapor pressure at free stream. The diffusion co-efficient (D_m) of vapor-NCG mixture is calculated from the Chapman-Enskog theory,[3]

$$D_m = 2.11 \times 10^{-5}\left(\frac{T_m}{273.15}\right)^{1.94}\left(\frac{1}{P_m}\right) \tag{S26}$$

Based on the mass conservation, Eq. S19, S20, S25 can be solved simultaneously. These equations consists of three unknown variables: $\dot{m}$, T_d and T_i. A Matlab script has been used for the computation of the unknown parameters by iterative solution of the equations Eq. S19, S20, S25. The heat transfer rate per droplet in presence of NCG is given by,

$$(q_d)_{NCG} = \dot{m}h_{lv} \tag{S27}$$

Figures 5D compare the magnitude of temperature drop across the droplet and vapor region for a condensate drop of radius 100 nm - 1 mm during the condensation of pure vapor and the vapor-NCG mixture. The figures are plotted for a moderate subcooling of 10 $°C$ with δ_{coat}= 1 μm, k_{coat}= 0.2 W/mK, θ= 90 °, k_l= 0.6 W/mK, h_{lv}= 2257 kJ/kg, σ_{lv}= 0.072 N/m, ρ_l= 997 kg/m^3, P_v=101.325 kPa, T_{sat}=373.15 K, v_v= 1.672 m^3/kg.[5]

Filmwise condensation

The total temperature difference (ΔT) between the cold surface and bulk vapor region during filmwise condensation can be expressed as,

$$\Delta T = \Delta T_{film} + \Delta T_{vapor} \tag{S28}$$

The temperature drop across the condensate film (ΔT_{film}) due to conduction is given by,

$$\Delta T_{film} = \frac{q''\delta_f}{k_l} \tag{S29}$$

where q'' is the average heat flux, δ_f is the average condensate film thickness approximated as ~25 μm.[13]

The average heat flux during pure vapor condensation is calculated from the Nusselt film condensation theory,[14]

$$q''_{pure\ vapor} = 0.943\left(\frac{g\rho_l(\rho_l - \rho_v)k_l^3 h'_{lv}}{\mu_l \Delta T L}\right)^{0.25} \Delta T \tag{S30}$$

where g is the acceleration due to gravity, $h'_{lv} = h_{lv} + C_p\Delta T$ in which C_p is heat capacity at a constant pressure, μ_l is the viscocity of the condensate film, L is length of the substrate.

The mathematical formulation of heat flux during water vapor condensation in the presence of NCG is challenging due to the existence of a gradient in vapor concentration and temperature above the condensate film. Therefore, previous research has proposed many empirical correlations to calculate the average heat flux during the filmwise mode of condensation in the presence of NCG.[4,15–17] In this analysis, the average heat flux for the condensation of water vapor from vapor-NCG mixture is approximated from the Uchida correlation,[17]

$$q''_{NCG} = U_{Uchida}\Delta T = 380\left(\frac{\omega_v}{1 - \omega_v}\right)^{0.7} \Delta T \tag{S31}$$

where U_{Uchida} is the overall condensation heat transfer co-efficient for the vapor-NCG mixture environment and ω_v is the percentage of vapor content in the mixture. Figure 5E is plotted using this analysis for a subcooling of 10 $°C$.

Supplementary Videos

Movie S1: Marco-scale condensation observation on superhydrophobic copper substrate for a duration of 15 *minutes*. The video is recorded a digital camera at 1 fps and the playback speed is $10X$.

Movie S2: Macro-scale condensation observation on superhydrophobic aluminum substrate for a duration of 15 *minutes*. The video is recorded using a digital camera at 1 fps and the playback speed is $10X$.

Movie S3: Illustration of condensate growth, rivulet formation, and drainage on superhydrophilic aluminum substrate for a duration of 1 *hour*. The video is recorded using an IR camera at 1 fps and the playback speed is $30X$.

Movie S4: Illustration of condensate growth, rivulet formation, and drainage on superhydrophilic copper substrate for a duration of 1 *hour*. The video is recorded using an IR camera at 1 fps and the playback speed is $30X$.

References

(1) Beysens, D. *The Physics of Dew, Breath Figures and Dropwise Condensation*; Springer International Publishing, 2022; Vol. 994; pp 25–35.

(2) Ranathunga, D. T.; Shamir, A.; Dai, X.; Nielsen, S. O. Molecular dynamics simulations of water condensation on surfaces with tunable wettability. *Langmuir* **2020**, *36*, 7383–7391.

(3) Jacobson, M. Z.; Jacobson, M. Z. *Fundamentals of atmospheric modeling*; Cambridge University Press, 1999.

(4) Das, C.; Gupta, R.; Halder, S.; Datta, A.; Ganguly, R. Filmwise condensation from humid air on a vertical superhydrophilic surface: Explicit roles of the humidity ratio difference and the degree of subcooling. *Journal of Heat Transfer* **2021**, *143*, 061601.

(5) Kim, S.; Kim, K. J. Dropwise condensation modeling suitable for superhydrophobic surfaces. *Journal of Heat Transfer* **2011**, *133*, 081502.

(6) Carey, V. P. *Liquid-vapor phase-change phenomena: An introduction to the thermophysics of vaporization and condensation processes in heat transfer equipment*; CRC Press, 2020.

(7) Tanasawa, I. *Advances in condensation heat transfer*; Elsevier, 1991; Vol. 21; pp 55–139.

(8) Luo, X.; Fan, Y.; Qin, F.; Gui, H.; Liu, J. A new model for the processes of droplet condensation and evaporation on solid surface. *International Journal of Heat and Mass Transfer* **2016**, *100*, 208–214.

(9) Zheng, S.; Eimann, F.; Philipp, C.; Fieback, T.; Gross, U. Modeling of heat and mass transfer for dropwise condensation of moist air and the experimental validation. *International Journal of Heat and Mass Transfer* **2018**, *120*, 879–894.

(10) Aoki, K.; Bardos, C.; Takata, S. Knudsen layer for gas mixtures. *Journal of Statistical Physics* **2003**, *112*, 629–655.

(11) Young, J. The condensation and evaporation of liquid droplets at arbitrary Knudsen number in the presence of an inert gas. *International Journal of Heat and Mass Transfer* **1993**, *36*, 2941–2956.

(12) Wen, R.; Zhou, X.; Peng, B.; Lan, Z.; Yang, R.; Ma, X. Falling-droplet-enhanced filmwise condensation in the presence of non-condensable gas. *International Journal of Heat and Mass Transfer* **2019**, *140*, 173–186.

(13) Zhang, W.; Wang, S.; Lianbo, M. Analytical modeling for vapor condensation in the presence of noncondensable gas and experimental validation. *Journal of Heat Transfer* **2021**, *143*, 011601.

(14) Nusselt, W. The surface condensation of water vapour. *Zeitschrift Des Vereines Deutscher Ingenieure* **1916**, *60*, 541–546.

(15) Tagami, T. *Interim report on safety assessments and facilities establishment project for June 1965, No. 1*; Japanese Atomic Energy Research Agency, 1965.

(16) Dehbi, A. A generalized correlation for steam condensation rates in the presence of air under turbulent free convection. *International Journal of Heat and Mass Transfer* **2015**, *86*, 1–15.

(17) Uchida, H.; Oyama, A.; Togo, Y. *Evaluation of post-incident cooling systems of light water power reactors*; Tokyo Univ., 1964.

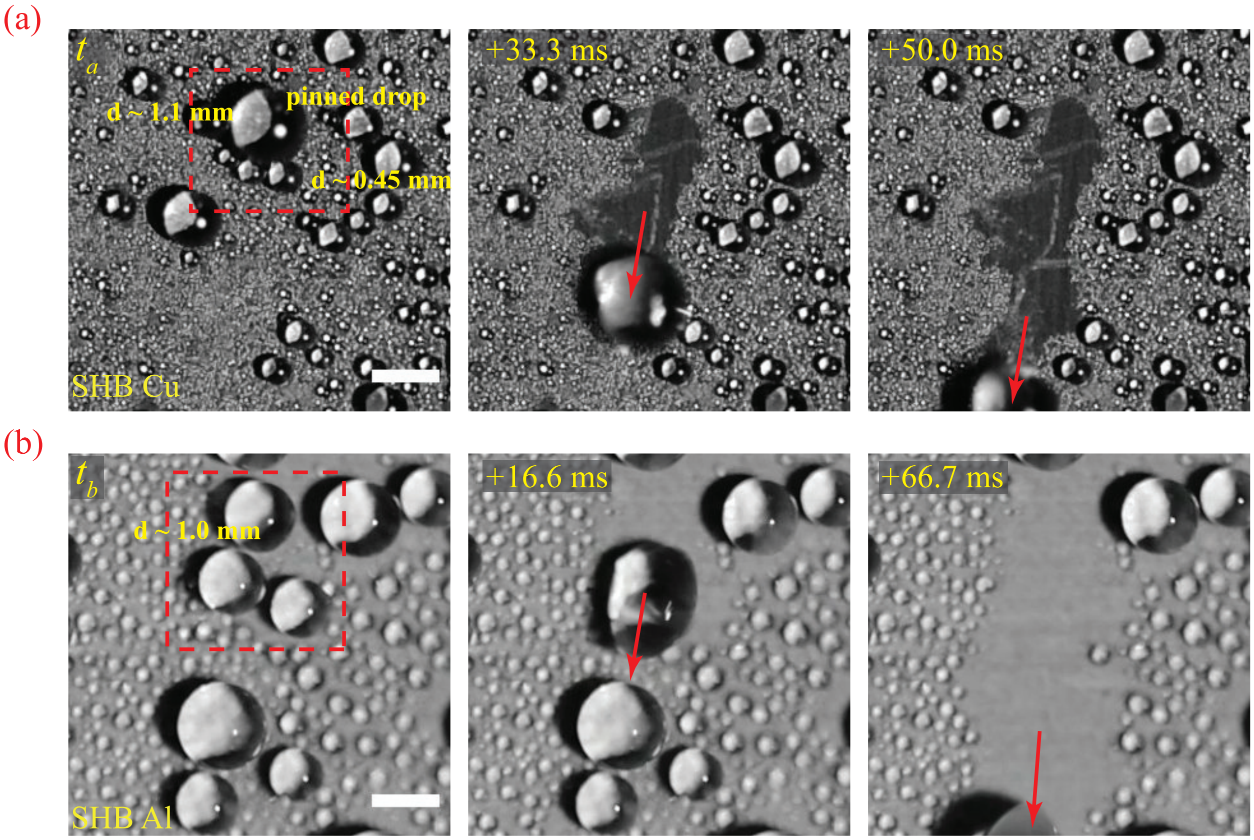

Figure S1: Condensate drainage from superhydrophobic surfaces. The sweeping mechanisms of (a) SHB copper substrate and (b) SHB aluminum substrate at T_{env}= 30 $°C$ and RH= 60 %. The scale bar is 1 mm.

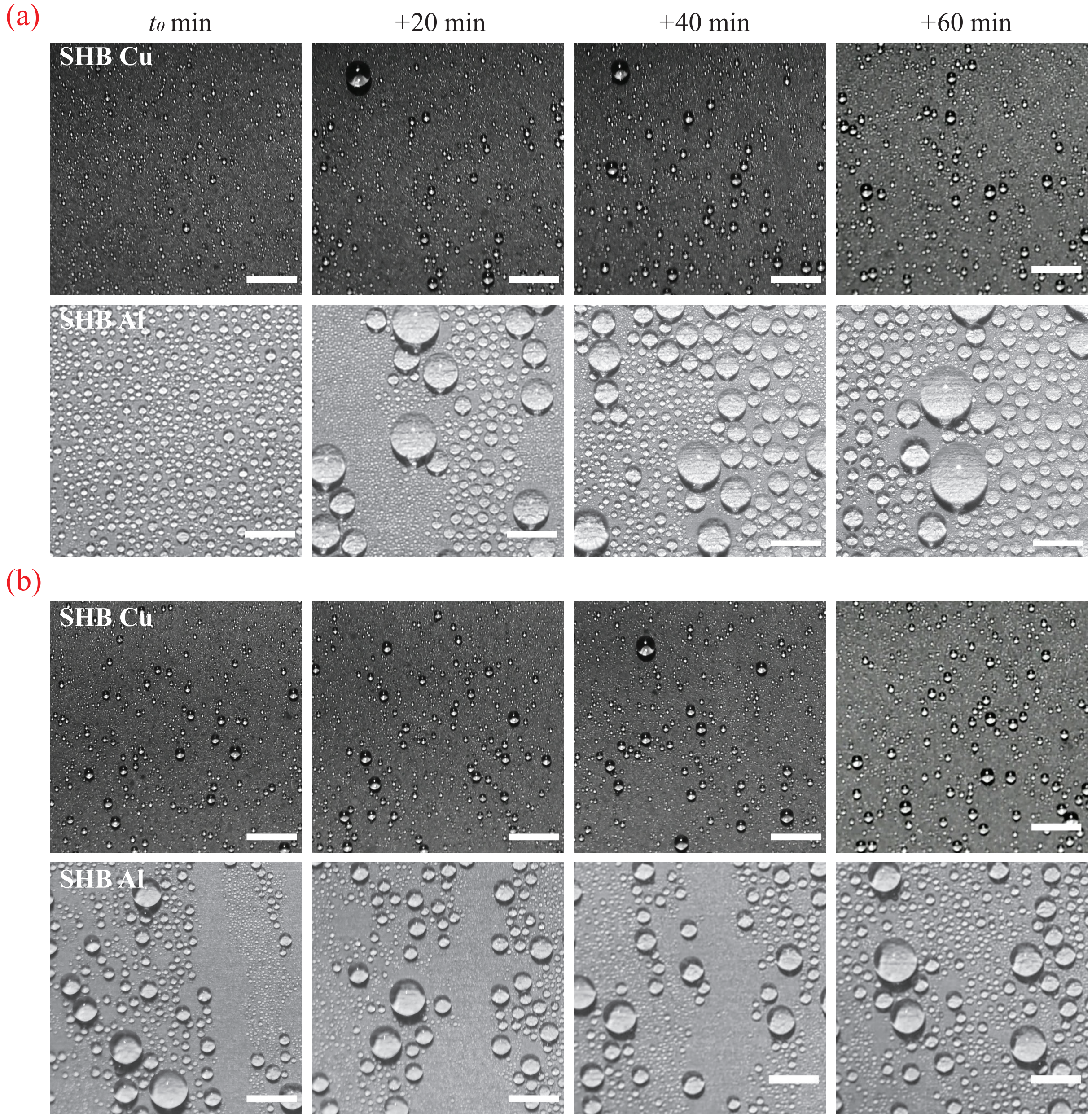

Figure S2: The observation of dropwise condensation at macro-scale. The time-lapse snapshots of condensation behavior on SHB copper and aluminum substrates at (a) T_{env}= 20 $°C$ and RH= 75 %, (b) T_{env}= 45 $°C$ and RH= 90 %. t_0 is the time at which the surface temperature reaches the set value. The scale bar is 2 mm.

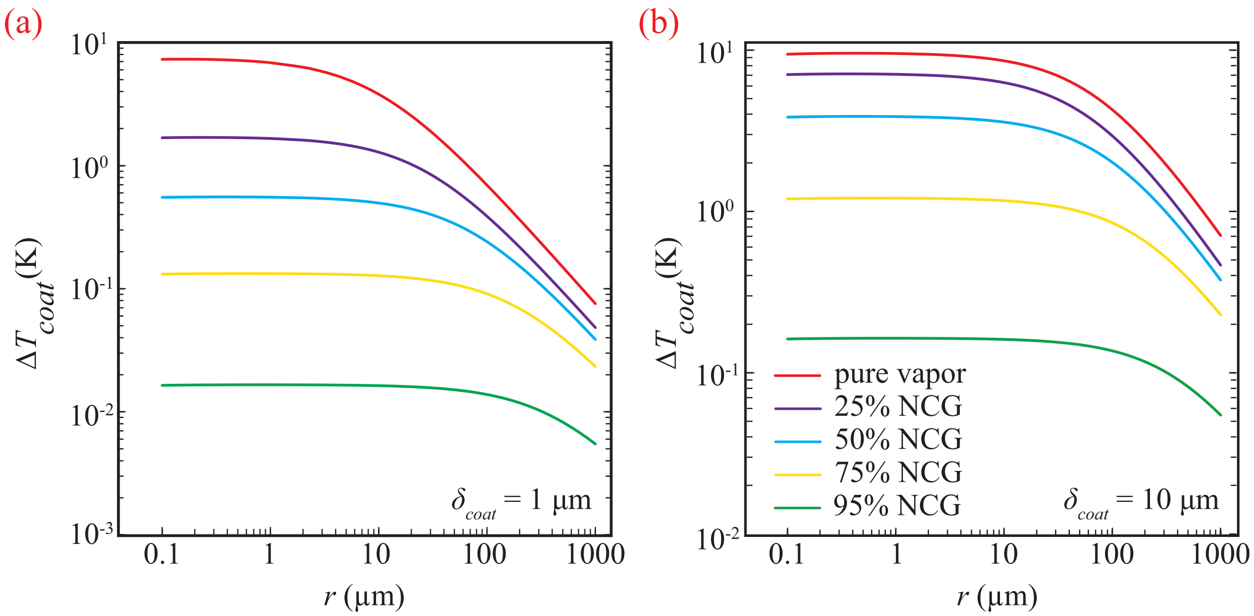

Figure S3: The temperature drop across the hydrophobic coating. The effect of NCG concentrations on the temperature drop across the hydrophobic promoter layer coating (ΔT_{coat}) of thickness (a) 1 μm, (b) 10 μm.

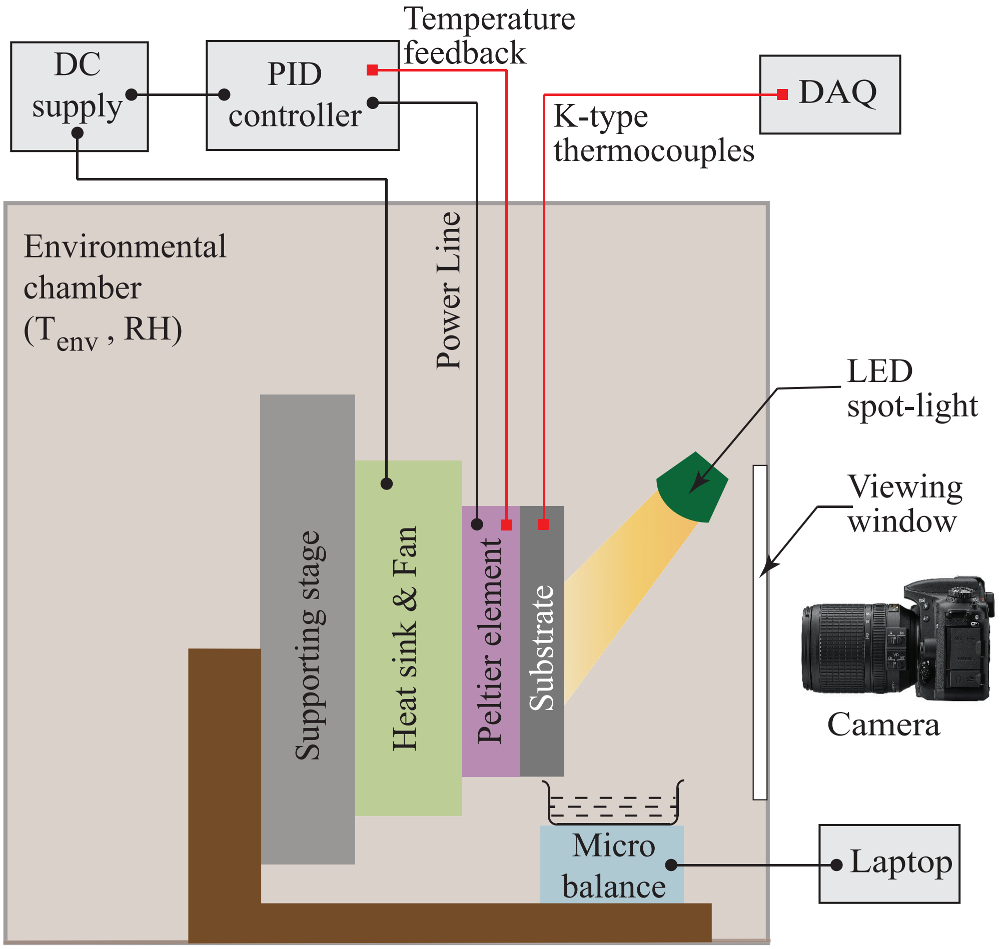

Figure S4: Schematics of the experimental setup. The condensation experiments were performed in a controlled environmental chamber. The temperature of the substrate was maintained at a constant temperature using a Peltier element.
